# Identifying Homelessness among Veterans Using VA Administrative Data: Opportunities to Expand Detection Criteria

**DOI:** 10.1371/journal.pone.0132664

**Published:** 2015-07-14

**Authors:** Rachel Peterson, Adi V. Gundlapalli, Stephen Metraux, Marjorie E. Carter, Miland Palmer, Andrew Redd, Matthew H. Samore, Jamison D. Fargo

**Affiliations:** 1 Salt Lake Informatics, Decision-enhancement and Analytic Sciences (IDEAS 2.0) Center, VA Salt Lake City Health Care System, Salt Lake City, Utah, United States of America; 2 Department of Psychology, Utah State University, Logan, Utah, United States of America; 3 Department of Internal Medicine, University of Utah School of Medicine, Salt Lake City, Utah, United States of America; 4 National Center on Homelessness Among Veterans, Philadelphia, Pennsylvania, United States of America; 5 Health Policy and Public Education, University of the Sciences, Philadelphia, Pennsylvania, United States of America; University of Illinois-Chicago, UNITED STATES

## Abstract

Researchers at the U.S. Department of Veterans Affairs (VA) have used administrative criteria to identify homelessness among U.S. Veterans. Our objective was to explore the use of these codes in VA health care facilities. We examined VA health records (2002-2012) of Veterans recently separated from the military and identified as homeless using VA conventional identification criteria (ICD-9-CM code V60.0, VA specific codes for homeless services), plus closely allied V60 codes indicating housing instability. Logistic regression analyses examined differences between Veterans who received these codes. Health care services and co-morbidities were analyzed in the 90 days post-identification of homelessness. VA conventional criteria identified 21,021 homeless Veterans from Operations Enduring Freedom, Iraqi Freedom, and New Dawn (rate 2.5%). Adding allied V60 codes increased that to 31,260 (rate 3.3%). While certain demographic differences were noted, Veterans identified as homeless using conventional or allied codes were similar with regards to utilization of homeless, mental health, and substance abuse services, as well as co-morbidities. Differences were noted in the pattern of usage of homelessness-related diagnostic codes in VA facilities nation-wide. Creating an official VA case definition for homelessness, which would include additional ICD-9-CM and other administrative codes for VA homeless services, would likely allow improved identification of homeless and at-risk Veterans. This also presents an opportunity for encouraging uniformity in applying these codes in VA facilities nationwide as well as in other large health care organizations.

## Introduction

U.S. Veterans are at higher risk for homelessness compared to the general population and are over-represented within the homeless population [1]. According to the U.S. Department of Housing and Urban Development [2], Veterans account for 9.5% of the U.S. adult population, yet represent 12% of the homeless adult population. Subsequently, the U.S. Department of Veteran Affairs (VA) has become the largest provider of health care services for homeless individuals in the U.S. [3]. Providing appropriate services to Veterans experiencing homelessness acutely depends on the VA health care system accurately recognizing them or those at risk as soon as possible. Those who are not promptly recognized or those who do not seek help (for various reasons) are not offered or are unable to access appropriate services to mitigate their circumstances.

By convention, VA researchers have used administrative criteria to identify homelessness among Veterans, such as ICD-9-CM coding by providers and VA specific administrative codes for outpatient clinics and inpatient treatment specialties indicating receipt of homelessness services [4–7]. The key element of the currently used criteria for identification of homelessness is an ICD-9-CM code of V60.0, signifying a lack of housing. However, additional ICD-9-CM codes in the V60 series (collectively referred to as V60.x in this study) exist with regard to housing instability (Table 1) [8].

**Table 1:** Comparison of V60 series ICD-9-CM codes and Z59 series ICD-10-codes from the Centers for Medicare & Medicaid Services. From http://www.cms.gov/medicare-coverage-database/staticpages/icd-9-code-lookup.aspx?KeyWord=v60&bc=AAAAAAAAAAAQAA%3d%3d&; Accessed November 1, 2014

ICD-9 CODE	ICD-9 CODE DESCRIPTION	ICD-10 CODE	ICD-10 CODE DESCRIPTION
**V60.0**	**LACK OF HOUSING**	**Z59.0**	**HOMELESSNESS**
**V60.1**	**INADEQUATE HOUSING**	**Z59.1**	**INADEQUATE HOUSING**
V60.2	INADEQUATE MATERIAL RESOURCES	Z59.2	DISCORD WITH NEIGHBORS, LODGERS AND LANDLORDS
V60.3	PERSON LIVING ALONE	Z59.3	PROBLEMS RELATED TO LIVING IN RESIDENTIAL INSTITUTION
V60.4	NO OTHER HOUSEHOLD MEMBER ABLE TO RENDER CARE	Z59.4	LACK OF ADEQUATE FOOD AND SAFE DRINKING WATER
V60.5	HOLIDAY RELIEF CARE	Z59.5	EXTREME POVERTY
V60.6	PERSON LIVING IN RESIDENTIAL INSTITUTION	Z59.6	LOW INCOME
V60.81	FOSTER CARE (STATUS)	Z59.7	INSUFFICIENT SOCIAL INSURANCE AND WELFARE SUPPORT
**V60.89**	**OTHER SPECIFIED HOUSING OR ECONOMIC CIRCUMSTANCES**	**Z59.8**	**OTHER PROBLEMS RELATED TO HOUSING AND ECONOMIC CIRCUMSTANCES**
**V60.9**	**UNSPECIFIED HOUSING OR ECONOMIC CIRCUMSTANCE**	**Z59.9**	**PROBLEM RELATED TO HOUSING AND ECONOMIC CIRCUMSTANCES, UNSPECIFIED**

There have been no systematic investigations of the administrative use and consequences of applying V60.0 as compared to one of the V60.x codes or the clinic and treatment codes to identify Veterans experiencing homelessness. Additionally, it is possible that non-uniform or non-standardized assignment of V60.0 to Veterans who are indeed homeless (e.g., received a VA homeless service, but never received a V60.0 code) and, conversely, the non-recognition of homeless Veterans who might receive a V60.x code instead of V60.0, could result in needy and eligible Veterans not being offered appropriate services in a timely manner. This in turn could lead to barriers to access to care and disparities among an already vulnerable population of Veterans. A related issue is that if V60 codes are not being used in a systematic or standardized way in the VA, this would have implications for arriving at accurate estimates of the prevalence of homelessness among Veterans. Finally, as stated by Brown, et al. [9], “…even though the [Veterans Health Administration] has committed numerous resources to both identify and eliminate homelessness among veterans, at present, there is no single code or identifier that is consistently used to indicate homeless status.”

Collectively, these issues underscore the need to better understand and standardize the use of all V60 codes. With anticipated transitions to the ICD-10 classification system [10], which includes the same ICD-9-CM code categories applicable to Veterans experiencing (V60.0) or at-risk (V60.x) of homelessness (Table 1), this is an opportune time to investigate the use of these codes and offer recommendations for their uniform use in the future. This investigation could also have implications for how other large managed health care systems understand and standardize the use of these same codes [11].

The purposes of this study are to 1) explore the use of V60 codes in the VA health care system for identifying homelessness among Veterans and 2) contribute to the accuracy of determining homelessness among Veterans in order to enhance prevention and early intervention efforts in the VA for Veterans experiencing or at-risk for homelessness. As a first step, we address the hypothesis that V60.x codes are used to designate homelessness among Veterans in a similar way that the V60.0 code is used (i.e., Are V60.0 and V60.x codes used interchangeably? Are there similar characteristics among Veterans receiving V60.0, V60.x codes, or homeless VA services?). Second, we examine the temporal course of the application of V60.0 and V60.x codes in order to understand administrative changes in homelessness (i.e., How often does receipt of a V60.0 code follow the receipt of a V60.x code, and vice versa?). Third, we assess the differences among Veterans receiving an ICD-9-CM V60 code and those who receive a non-ICD designation (VA clinic or treatment specialty code) as their first indicator of homelessness (i.e., What proportion of Veterans designated as homeless via administrative records did not receive a ICD-9-CM V60 code as their first indicator, and how do they differ in terms of their demographic and VA service use characteristics?). Fourth, we explore rates of V60.0 and V60.x codes across VA facilities to determine whether these codes are used systematically (i.e., Do frequencies of V60.0 or V60.x codes vary by region?).

## Materials and Methods

### Veteran Cohort

VA records were examined for all individuals listed on an official roster indicating they had served in the U.S. military as part of the recent conflicts in Iraq or Afghanistan. Specifically, these include those who were deployed as part of Operation Enduring Freedom (OEF), Operation Iraqi Freedom (OIF), and Operation New Dawn (OND). This roster consisted of 941,970 Veterans who separated from the military after deployment as part of OEF/OIF/OND and who were eligible for and enrolled in VA health care services as of July 2011. Of these, 845,593 (89.8%) had at least one documented encounter within the VA system as of April 2012. Records from this latter group were taken from a nationwide VA research database of administrative and clinical data managed by Veterans Informatics and Computing Infrastructure (VINCI) [12] to ascertain homelessness.

Veterans were selected for analysis if they belonged to one of three mutually exclusive homeless groups, based on the *first* administrative record with any of the following homelessness-related indicators: 1) V60.0, 2) V60.x, and 3) non-ICD indicators of homelessness. The first category included all those with records of receiving a V60.0 ICD-9-CM code as part of a VA contact. The second category included those who received any of three V60.x series ICD-9-CM codes related to housing circumstances: V60.1 (inadequate housing), V60.89 (other specified housing or economic circumstances) and V60.9 (unspecified housing or economic circumstances) (see Table 1). The final category included those whose records contained at least one of a set of specific non-ICD VA clinic codes or treatment specialty codes[7]. These include VA-specific codes that designate the clinic where the Veteran received a particular homeless service:

1. 522 (Department of Housing and Urban Development VA Shared Housing [HUD-VASH]);
2. 528 (Telephone/Homeless Mentally Ill [HMI]);
3. 529 (Healthcare for Homeless Veterans);
4. 530 (Telephone/HUD-VASH); and
5. 590 (Community outreach to homeless Veterans by staff)

as well as the inpatient treatment specialty codes (homeless services for hospitalized Veterans):

1. 37 Domiciliary care for homeless Veterans (DCHV); and
2. 28 Mental Health Residential and Rehab Treatment Program for Compensated Work Therapy/Treatment Resident (MH RRTP CWT/TR).

Only Veterans who had at least one additional VA visit in the 90 days immediately following the identification of homelessness were retained in the sample to allow for an examination of receipt of at least one service (and associated ICD-9-CM codes) after the initial homeless designation. Outpatient clinic visits in the 90 days immediately after the first indicator of homelessness were categorized as either homeless, mental health, or substance abuse/addiction services. Additionally, comorbidities among VA homeless groups were also explored by summarizing their ICD-9-CM diagnoses. VA medical center region and station identification was also obtained from the administrative data.

### Data Analyses

Descriptive statistics for study variables were computed and stratified by VA homeless group (V60.0, V60.x, and non-ICD indicators). We computed the percentage of Veterans receiving 1) V60.0, V60.x or a non-ICD indicator of homelessness in a VA medical facility *after* separation from the military; 2) a V60.x code first and a *subsequent* V60.0 code within the following 90 days and 3) a V60.0 code first and a *subsequent* V60.x code within the following 90 days. We then analyzed the type of clinical service assigning either V60.0 or V60.x (using chi-square statistics). We next explored various outpatient homeless, mental health, and substance abuse services the Veterans utilized in the immediate 90 days after receiving a homeless designation, stratified by first indicator of homelessness group (e.g., V60.0, V60.x, non-ICD).

To investigate differences among Veterans receiving a V60.0, V60.x, or non-ICD designation as their *first* indicator of homelessness, we conducted a series of three binary logistic regression analyses with the following dependent variables: 1) V60.x compared to V60.0; 2) V60.x compared to the non-ICD indicator; and 3) V60.0 or V60.x (any V60) compared to the non-ICD indicator. The following covariates were included in all logistic regression models: sex, race (Black, White, Hispanic, Other/Unknown), marital status (married, never married, divorced/separated), level of education (high school or less, post-high school), rank (officer, enlisted), and VA evidence of mental health visits (yes, no) or receipt of substance abuse/addiction services (yes, no) in the 90 days following the first indicator of homelessness.

To explore the possibility of geographic variations in the use of specific V60 codes, we performed both cartographic and statistical modeling. Nationally, the VA healthcare system is administratively divided into four regions, which are further subdivided into 21 Veterans Integrated Service Network (VISN) areas that contain 152 separate medical facilities (stations). First, maps were generated for the 1) 21 VISNs and 2) 152 VA station areas for each fiscal year (2002–2012). These maps depicted the proportion of Veterans receiving each V60 code (V60.0, V60.1, V60.89, V60.9) of the total number of Veterans identified as homeless in each area. We then conducted statistical analyses to determine whether V60.0 and V60.x codes varied at the region, VISN, or medical facility level nationally. These analyses consisted of a set of multinomial models with three sets of predictor variables (region, VISN, and station) with the outcome being the proportion of Veterans with either V60.0 or V60.x.

Descriptive statistics and logistic regression analyses were conducted using SPSS Version 21 [13]. The geographic statistical models were fit using R version 3.0.2 [14] and the *multinom* function from the *nnet* package [15]. Maps were generated using ArcGIS [16].

### Ethics Statement

The work described was approved by the University of Utah and Utah State University Institutional Review Boards as well as by the Research and Development committee at VA Salt Lake City Health Care System. A waiver of authorization was approved for retrospective review of existing medical record data. All data was de-identified prior to analysis.

## Results

Of the 845,593 OEF/OIF/OND Veterans who had at least one documented encounter within the VA system as of April 2012, the current VA case definition of homelessness (V60.0 or homelessness-related clinic stop and specialty codes) was the basis for identifying 21,021 homeless Veterans (2.5%). Expanding the criteria to include the three V60.x codes increased that number to 31,260 Veterans (3.3% overall; 3.3% for males; 3.7% for females). Of these, 30,886 Veterans (98.8%) had at least one VA visit in the 90 days following the first identification of homelessness (using any of the aforementioned indicators) and were retained for further analysis.

A summary of the demographic characteristics of each “first indication of homelessness” group is presented in Table 2. Forty-two percent (n = 13,066) had V60.0 as their first indicator of homelessness, followed by 32.0% (n = 9,865) with V60.x (exclusive of V60.0). The remainder of the Veterans identified as homeless (n = 7,955, 25.8%) showed an initial receipt of a homeless service without having either type of V60-code in their record. The cohort was predominantly male (86.8%), White (42.3%), with many of “other” or “unknown” race (29.0%), and consisted almost exclusively of enlisted personnel (98.6%). The distributions of all demographic variables differed significantly following chi-square tests across 1^st^ homelessness classification groups due to the large sample sizes (all p < .0001).

**Table 2:** Characteristics of Veterans of Operations Enduring Freedom/Iraqi Freedom/New Dawn (OEF/OIF/OND) according to “first indicator of homelessness” group during fiscal years 2001–2012; N = 30,866. Note. All demographic variables differed significantly across 1^st^ homelessness classification groups following chi-square tests, all p < .0001.

	Total Sample		V60.0 1st Indicator		V60.x 1st Indicator		Non-ICD 1st Indicator	
	(N = 30,886, 100%)		(N = 13,066, 42.3%)		(N = 9,865, 32%)		(N = 7,955, 25.8%)	
Demographic Variables	N	%	N	%	N	%	N	%
Sex								
Male	26,818	86.8	11,399	87.2	8,425	85.4	6,994	87.9
Female	4,067	13.2	1,667	12.8	1,439	14.6	961	12.1
Missing	1	0.0	0	0.0	1	0.0	0	0.0
Race								
White	13,079	42.3	5,329	40.8	4,392	44.5	3,358	42.2
Black	5,490	17.8	2,484	19.0	1,751	17.7	1,255	15.8
Hispanic	3,374	10.9	1,279	9.8	1,125	11.4	970	12.2
Other/Unknown	8,943	29.0	3,974	30.4	2,597	26.3	2,372	29.8
Marital Status								
Married	11,119	36.0	4,405	33.7	3,830	38.8	2,884	36.3
Never Married	18,391	59.5	8,085	61.9	5,584	56.6	4,722	59.4
Divorced/Separated	1,349	4.4	560	4.3	445	4.5	344	4.3
Missing	17	0.1	16	0.1	6	0.1	5	0.1
Education								
Post-Secondary Education	3,072	9.9	1,131	8.7	1,038	10.5	903	11.4
High School Diploma or Less	27,427	88.8	11,757	90.0	8,702	88.2	6,968	87.6
Missing	99	1.3	178	1.4	99	1.3	7,871	98.9
Military Service								
Active Duty	19,960	64.6	8,706	66.6	6,236	63.2	5,018	63.1
Reserve	10,926	35.4	4,360	33.4	3,629	36.8	2,937	36.9
Rank								
Officer	442	1.4	143	1.1	150	1.5	149	1.9
Enlisted	30,444	98.6	12,923	98.9	9,715	98.5	7,806	98.1

Table 3 presents the frequency of each indicator of first instance of homelessness group, as well as for each specific category included in each group. Focusing only on V60 codes as the first indicator of homelessness using the expanded criteria, 57.0% received V60.0 and 43.0% received V60.x. In total, 18.4% (n = 3,280) of those receiving V60.x as their first indicator subsequently received V60.0 code within 90 days. Of those who first received V60.0, 1,303 individuals (10%) subsequently received a V60.x code in the following 90 days. Of those Veterans who received either V60.0 or V60.x, less than half (41%) were noted to have received a homeless service through the VA in the 90 days after receipt of the V60.0 code. However, a majority (81.3%) received mental health services and nearly a quarter (23.7%) received substance abuse services in that 90-day period.

**Table 3:** Frequency of first administrative indicator of homelessness *and* receipt of V60.0 and outpatient services in the 90 days immediately following the first administrative indicator among a cohort of OEF/OIF/OND Veterans during fiscal years 2001–2012. Note. ICD-9 codes V60.0 (lack of housing), V60.1 (inadequate housing), V60.89 (other specified housing or economic circumstance), V60.9 (unspecified housing or economic circumstances) and clinic stop codes 522 or 530 (Department of Housing and Urban Development VA shared housing program [HUD-VASH]), 528 (telephone/homeless mentally ill [HMI]), 529 (health care for homeless Veterans); 590 (community outreach to homeless Veterans), or inpatient homeless stays (28, homeless compensated mental health residential and rehab treatment program [(MH RRTP CWT/TR, treatment specialty]; 37, domiciliary care for homeless Veterans [DCHV, treatment specialty]).

			Within 90 Days Immediately after First Administrative Indicator of Homelessness							
	First Administrative Indicator of Homelessness		Subsequent receipt of V60 Designation		Homeless Services		Mental Health Services		Substance Abuse Services	
Homelessness Designation	N	%	N	%	N	%	N	%	N	%
**V60.0**	**13,066**	**42.3**	-	-	6,352	48.6	10,846	83.0	3,826	29.3
**V60.x**	**9,865**	**32.0**	1,794	18.2	3,041	30.8	7,793	79.0	1,611	16.3
* V60*.*1*	2,124	6.9	565	26.6	-	-	-	-	-	-
* V60*.*89*	3,989	12.9	674	16.9	-	-	-	-	-	-
* V60*.*9*	3,752	12.1	555	14.8	-	-	-	-	-	-
**Any V60** (V60.0 or V60.x)	**22,931**	**74.3**	-	-	9,393	41.0	18,639	81.3	5,437	23.7
**Any Non-ICD Indicator**	**7,955**	**25.7**	1,486	18.7	2,830	35.6	5,932	74.6	1,860	23.4
* Clinic Stop Code Indicating Receipt of Homeless Service*	7,588	24.6	1,290	17.0	-	-	-	-	-	-
* Inpatient Stay Indicating Homelessness*	367	1.2	196	53.4	-	-	-	-	-	-
**Total**	**30,866**	**100.0**	**3,280**	**10.6**	**12,223**	**39.6**	**24,571**	**79.6**	**7,297**	**23.6**

The frequency with which each specific type of service provider assigned V60.0 or V60.x for the first time for a given Veteran is presented in Table 4. Homeless service providers were the most common assignees of both V60.0 and V60.x codes, and, while V60.0 was assigned significantly more frequently than V60.x codes, almost 30% of the latter group received homeless services. Compared to the assignment of V60.0, V60.x codes were administered 1) significantly more frequently in medical specialty, primary care, and social work settings (all p < 0.001); 2) significantly less frequently in alcohol/substance abuse, emergency department, homeless, and inpatient settings (all p < 0.0001); and 3) with no difference in surgical specialties, mental health, and lab/imaging settings (all p > 0.05).

**Table 4:** Type of service providers *assigning* V60 codes to administratively code for homelessness for the first time among a cohort of OEF/OIF/OND Veterans, 2001–2012. Note. Values represent N (%). χ2 test and p-value come from z-tests for independent proportions with a correction for continuity.

Service Provider Type	V60.0 Code	V60.x Code	χ2	p
	N (%)	N (%)		
Homeless Services	4,525 (34.6)	2,874 (29.1)	77.51	<0.0001
Social Work	2,885 (22.1)	2,891 (29.3)	155.35	<0.0001
Primary Care	1,742 (13.3)	1,470 (14.9)	11.36	0.0008
Lab/Imaging	1,314 (10.1)	923 (9.4)	3.05	0.0806
Mental Health Providers	1,317 (10.1)	927 (9.4)	2.89	0.0891
Inpatient	303 (2.3)	132 (1.3)	28.54	<0.0001
Alcohol/Substance Abuse Services	285 (2.2)	128 (1.3)	24.32	<0.0001
Medical Specialties	258 (2.0)	325 (3.3)	38.99	<0.0001
Unknown	198 (1.5)	108 (1.1)	7.24	0.0071
Emergency Department	151 (1.2)	45 (0.5)	31.64	<0.0001
Surgical Specialties	20 (0.2)	12 (0.1)	0.21	0.6509
Research	3 (0)	1 (0)	NA	NA
**Total (All Provider Types)**	**13,066 (100)**	**9,865 (100)**		

In the 90 days immediately after the first indicator of homelessness, Veterans receiving V60.0 logged an average of 14 visits (median 9, range 1–90) to VA medical facilities, similar to those with V60.x (average 11, median 7, range 1–89) (Table 5). Veterans receiving V60.0 or V60.x as their first indicator appeared to have a similar profile in terms of their co-morbidities (as shown by the different categories of ICD-9-CM codes assigned to their visit) (Table 5).

**Table 5:** Frequencies of ICD-9-CM codes assigned to VA medical visits for a cohort of OEF/OIF/OND Veterans with an administrative indicator of homelessness (in the 90 days immediately after the first administrative indicator).

ICD-9-CM Categories (clinical classifications software from http://www.hcup-us.ahrq.gov/toolssoftware/ccs/ccs.jsp)	V60.0 Code		V60.x Code	
	Total Veterans: 13,066 Total Visits: 188,151 Average visits: 14 Median: 9 Range: 1–90		Total Veterans: 9,875 Total Visits: 105, 556 Average visits: 11 Median: 7 Range: 1–89	
	N	(% of all codes)	N	(% of all codes)
Symptoms; signs; and ill-defined conditions and factors influencing health status	11863	23	8271	22
Mental Illness (including substance abuse/dependency)	10559	21	7516	20
Diseases of the musculoskeletal system and connective tissue	5066	10	4070	11
Diseases of the nervous system and sense organs	4070	8	3157	8
Residual Codes (Unclassified)	3074	6	2195	6
Infectious and Parasitic Diseases	3045	6	1822	5
Diseases of Digestive System	2903	6	2080	6
Injury and Poisoning	2357	5	1838	5
Endocrine; nutritional; and metabolic diseases and immunity disorders	2231	4	1744	5
Diseases of the Circulatory system	1794	3	1447	4
Diseases of the Respiratory system	1743	3	1219	3
Skin and Subcutaneous Tissue Infections	1132	2	831	2
Diseases of the Genitourinary system	882	2	764	2
Neoplasms	254	0	287	1
Complications of pregnancy; childbirth; and the puerperium	248	0	221	1
Diseases of the blood and blood-forming organs	213	0	176	0
**Total number of ICD-9-CM codes**	**51434**		**37638**	

Table 6 summarizes the results of three binary logistic analyses regressing first indicators of homelessness on demographic, military service, and health-related covariates: 1) V60.x versus V60.0; 2) V60.x versus non-ICD indicator; and 3) any V60 code (V60.0 or V60.x) versus non-ICD indicator. Results of the first model indicated that several Veteran characteristics were significantly associated (all p < 0.05) with a higher likelihood of receiving a V60.x as compared to a V60.0 designation: being male, Black or of other/unknown race/ethnicity, never married, having a high school education or less, served as an active duty service member, and not having received mental health or substance abuse services. Several Veteran characteristics were significantly associated with a higher likelihood of receiving V60.x as compared to a non-ICD indicator designation in the second model: being male, Black or Hispanic, a reservist, and receiving mental health, but not substance abuse, services. The results of the third model showed that several Veteran characteristics were significantly associated with a higher likelihood of receiving any V60 code as the first indicator of homelessness compared to a non-ICD indicator: being male, non-White (Black, Hispanic, or Other/Unknown), having a post-secondary education, served in the reserves, and received mental illness services.

**Table 6:** Results of logistic regression analyses of first administrative indicator of homelessness classification. Note. OR = odds ratio, CI = confidence interval.

		V60.x vs. V60.0		V60.x vs. Non-ICD indicator		Any V60 (.0 or.x) Code vs. Non-ICD indicator	
Variable		O.R.	95% C.I.	O.R.	95% C.I.	O.R.	95% C.I.
**Sex** (Ref = Male)	Female	1.14***	1.05, 1.23	1.21***	1.10, 1.32	1.12**	1.04, 1.22
**Race**	Black	0.79***	0.74, 0.85	0.82***	0.76, 0.88	0.93*	0.88, 1.00
(Ref = White)	Hispanic	1	0.92, 1.09	0.79***	0.72, 0.87	0.80***	0.73, 0.86
	Other/Unknown	0.79***	0.72, 0.87	0.96	0.87, 1.07	1.10*	1.01, 1.20
**Marital Status**	Never Married	0.86*	0.75, 0.99	0.96	0.83, 1.12	1.05	0.92, 1.20
(Ref = Married)	Divorced/Separated	1.3	0.90, 1.18	1.08	0.93, 1.25	1.06	0.93, 1.21
**Education** (Ref = Post- Secondary Educ.)	No Post-Secondary Educ.	0.89*	0.81, 0.98	1.09	0.98, 1.21	1.16***	1.06, 1.27
**Rank** (Ref = Officer)	Enlisted	0.9	0.70, 1.16	1.16	0.90, 1.49	1.23	0.99, 1.52
**Active Duty/Reserve** (Ref = Active Duty)	Reserve	1.09**	1.02, 1.15	0.93*	0.87, 1.00	0.90***	0.85, 0.96
**Mental Illness** (Ref = No Mental Illness services received)	Receipt of Mental Illness Services	1.15***	1.07, 1.23	0.72***	0.67, 0.78	0.67***	0.63, 0.71
**Substance Abuse** (Ref = No Sub. Abuse services received)	Receipt of Substance Abuse Services	2.07***	1.94, 2.21	1.65***	1.53, 1.78	1.05	0.99, 1.12

* p < 0.05.

** p < 0.01

*** p < 0.00

**Fig 1:**
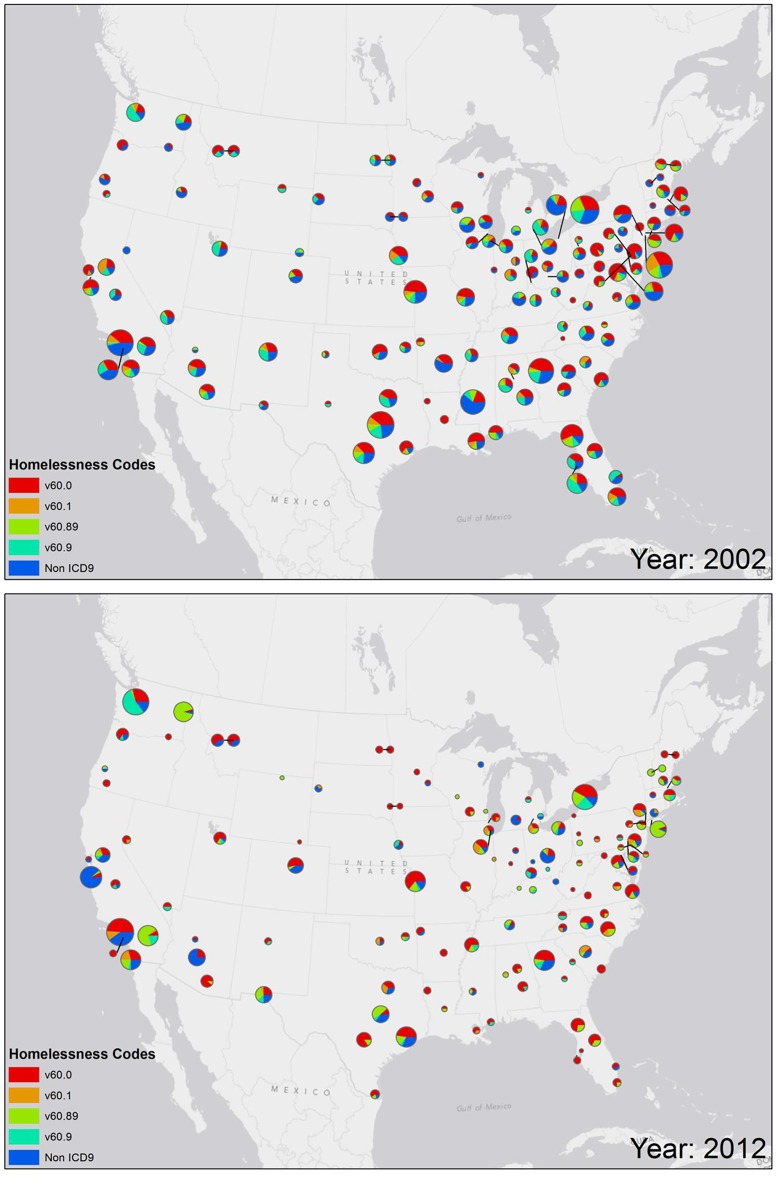
Map of usage of various administrative indicators for the recognition of homelessness in VA medical facilities across the country (V60.0, V60.1, V60.89. V60.9 and non-ICD codes: clinic stop codes and inpatient treatment codes as described in the text). The pie charts are derived from a normalized ratio: the frequency of the specific code given as a first indicator of homelessness (numerator) as a proportion of the total number of Veterans identified as homeless in the area (denominator). Two representative years are shown.

**Fig 2:**
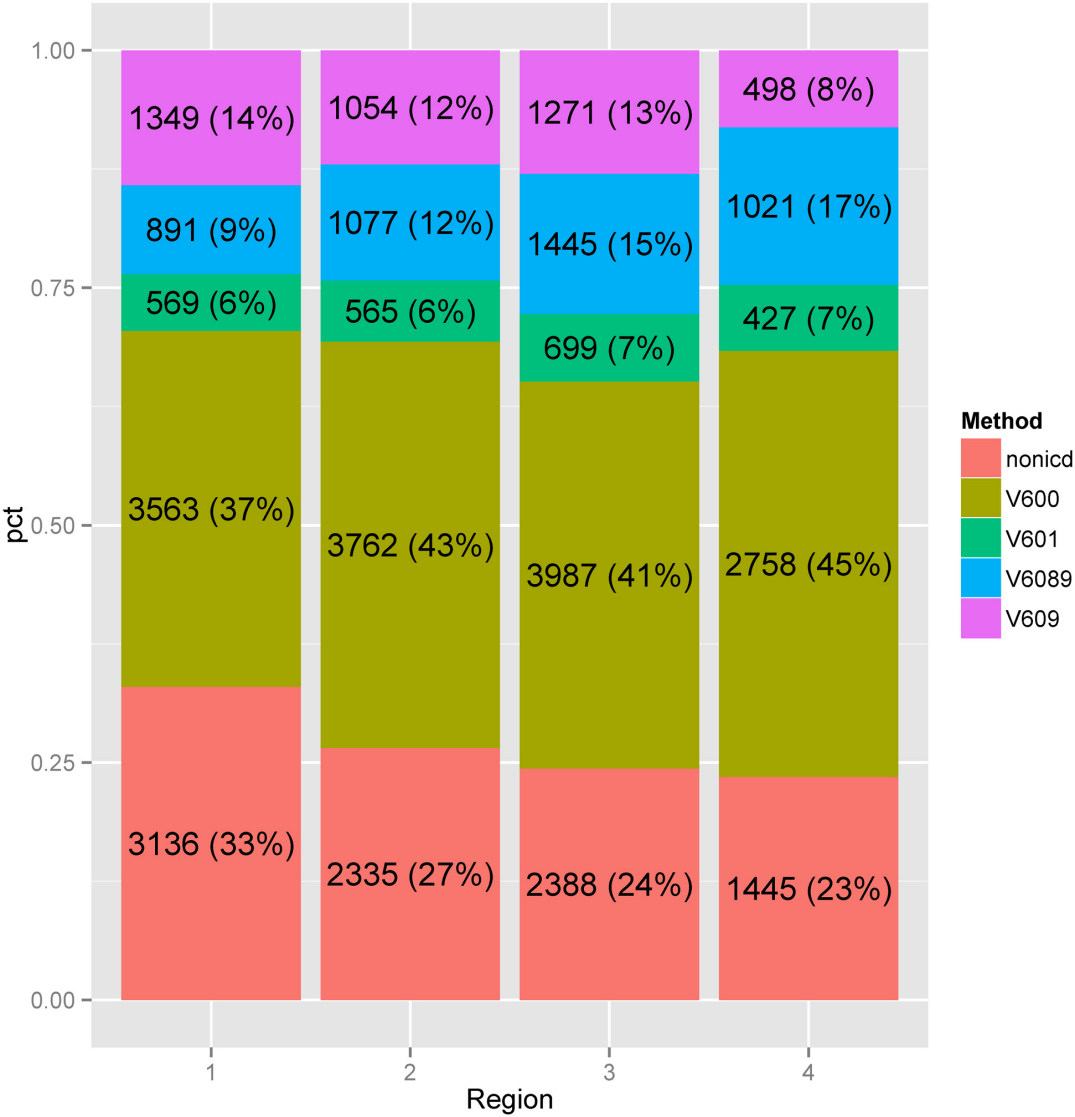
A stacked bar graph of the variability in usage of V60 and non-ICD codes to identify homelessness among Veterans across 4 VA regions in the US. Region 1 includes the East Coast, Region 4 the West Coast. The time period studied is 2002–2012.

As shown on the map in Fig 1 (snapshot of 2002 and 2012 data for station level geographies), there tended to be a greater than average use of V60.0 in VA medical facilities in the coastal areas of the country; whereas in VA facilities in the central parts of the U.S., there was less-than-average use of the V60.0 code. An animation of these maps across the 10-year period, available in the (S1 File), shows a similar trend in the use of V60 and non-ICD codes at VA stations around the country. Results of the multinomial models showed statistically significant differences (p < 0.001) in the use of V60.0 versus V60.x across all geographic levels (region, VISN and station), indicating differences in the pattern of usage of these codes around the country at the region, VISN, and station level. There was a progressive increase in the use of V60.0 from Region 1 (East Coast) to Region 4 (West Coast); a reciprocal usage pattern of V60.89 and V60.9, and a decrease in use of non-ICD codes (Fig 2).

## Discussion

These findings suggest that identifying homelessness based upon VA administrative records alone may lead to challenges in determining the extent of this problem among Veterans seeking care in VA medical facilities. Inferring homelessness based on a combination of V60 codes (from the ICD-9-CM) and evidence of receipt of homeless-related VA services is sensitive to the criteria used. The conventional criteria (V60.0 code or a non-ICD-9-CM administrative code for receipt of VA homeless services) identified 2.5% of Veterans who served in Iraq or Afghanistan as experiencing homelessness. When adding an additional set of V60 codes (denoted as V60.x in this study), which expands these criteria to include conditions indicating unstable housing, this proportion increased to 3.3%. Many of these added by a V60.x code subsequently also received a V60.0 code or a non-ICD-9-CM administrative code.

It is likely that, in many instances, V60.x codes are either being used interchangeably with V60.0 by VA staff for indicating homelessness or that Veterans so designated have an unstable housing situation and subsequently become homeless. Determining which explanation is more prevalent goes beyond the parameters of these data, but the data do indicate fluidity among the expanded set of V60 codes and the conventional criteria for determining homelessness. It is likely that this fluidity is, in part, due to Veterans moving interchangeably from housing instability (V60.x) to literal homelessness (V60.0) as they negotiate a series of makeshift housing arrangements. This situation of assigning administrative codes for homelessness creates a degree of ambiguity that will likely persist after billing and coding systems are converted to ICD-10 in the U.S. (now scheduled for October 2015), as the equivalent ICD-10 codes for homelessness (Z59 series, Table 1) are similar to those in ICD-9-CM.

Though there appears to be some variability in which type of provider assigns these codes and variation in the use of these codes across VA facilities, the co-morbidity profile of these Veterans appears to be similar. It was also interesting to note that over half of the Veterans in this OEF/OIF/OND cohort who received a V60.0 or V60.x code did not have a record of receiving a VA homeless service in the 90 days after receiving the code. Automated follow-up procedures should be in place to see that all cases of identified housing instability, whether based on V60-codes or on more direct ways of determining homelessness, are provided the opportunity to connect with VA homeless services.

The strengths of our study include the availability of longitudinal medical records of military service members on the OEF/OIF/OND roster who subsequently received care in VA medical facilities over a 10-year period and the nationally representative samples of homeless Veterans from that era. The numbers of homeless Veterans analyzed here are among the largest reported in the literature.

We acknowledge several limitations. The reliability and usage of the ICD-9-CM codes analyzed (V60.0 and V60.x) may have changed over time from 2001 to 2012 and may represent a surveillance artifact. Other than the administrative indicators of homelessness and subsequent homeless/allied service use, for this study, we have not independently verified the homeless status of Veterans by interviewing Veterans or by a manual review of medical notes. There is a need for further studies to validate the use of V60 codes for homelessness in the VA with detailed follow-up and review of electronic medical records. Our analyses are restricted to those Veterans seeking care in the VA; we have no data on those Veterans who may be homeless and have never sought care in the VA (for any reason, including ineligibility for VA services).

In conclusion, these results support the creation of a standard administrative definition and method for identification of homeless Veterans. The expanded V60.x criteria proposed here would be consistent with changes in the 2009 HEARTH Act [17], in which the federal definition of homelessness broadened to encompass imminent loss of housing (within 14 days with no subsequent residence identified and a lack of resources or support to obtain permanent housing).

With the VA being the largest healthcare provider for individuals experiencing homelessness [3], our findings present an opportunity for encouraging uniformity in applying these codes now as well as in the future as VA transitions to ICD-10 in 2015. The challenges of using administrative data alone for determining homelessness may be mitigated by the recent introduction of a screener for homelessness among Veterans [18] or the use of informatics technologies to identify homelessness from the written medical record [19,20].

Our work in the VA likely has application to large health care organizations (inner city hospitals, academic centers, Medicaid managed care organizations) that provide care to individuals experiencing homelessness. Providers in VA medical facilities across the country, especially those in primary care, would likely benefit from an increased awareness of homelessness among Veterans and the importance of appropriate ‘coding’ to trigger the initiation of appropriate services.

## Supporting Information

S1 File Time lapse animation showing maps of the U.S. and the usage of V60.x codes by VA providers over a 10-year period (2002–2012). There tended to be a greater than average use of V60.x codes in VA medical facilities in the coastal areas of the country; whereas in VA facilities in the central parts of the U.S., there was less-than-average use of the V60.0 code.
(GIF)
