# Supplementary figures and images for: Identifying Homelessness among Veterans Using VA Administrative Data: Opportunities to Expand Detection Criteria
Source: PLoS One. 2015 Jul 14;10(7):e0132664. doi: 10.1371/journal.pone.0132664 (PMC4501742; doi:10.1371/journal.pone.0132664)

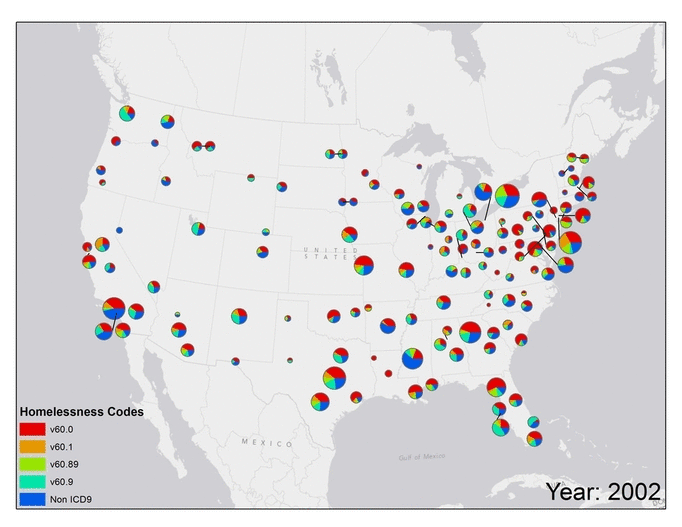

Supplement: S1 File — There tended to be a greater than average use of V60.x codes in VA medical facilities in the coastal areas of the country; whereas in VA facilities in the central parts of the U.S., there was less-than-average use of the V60.0 code. (GIF) [file pone.0132664.s001.gif]
